# Overwinter Changes in the Lipid Profile of Young-of-the-Year Striped Bass (*Morone saxatilis*) in Freshwater Ponds

**DOI:** 10.3390/biom11111678

**Published:** 2021-11-11

**Authors:** Kare A. Tonning, Suzanne M. Budge, Peter Tyedmers

**Affiliations:** 1 School for Resource and Environmental Studies, Dalhousie University, Halifax, NS B3H 4R2, Canada; Peter.Tyedmers@dal.ca; 2 Process Engineering and Applied Science, Dalhousie University, Halifax, NS B3H 4R2, Canada; Suzanne.Budge@dal.ca

**Keywords:** thermal acclimation, endogenous reserves, phospholipid, *Morone saxatilis*

## Abstract

Young-of-the-year (YOY) striped bass (*Morone saxatilis*) suffer significant mortality during their first winter. While causes of this mortality are unclear, lipids may play role in adapting to winter stresses, including thermal change and food scarcity. To address this, YOY striped bass were placed in mesh cages in freshwater ponds in the fall (November) and were held until the end of winter, in March. Liver and white muscle tissue were sampled at the beginning and end of the study to compare concentrations of specific lipid classes and fatty acid composition. Muscle-tissue total lipid and triacylglycerol (TAG) was higher in March (late winter) samples. Additionally, concentrations of phosphatidylethanolamine (PE) were higher in the white muscle of striped bass sampled in March; this was accompanied by a decrease in proportions of 18:0 and 22:6n-3 in PE (from ~11 to 7% and 36 to 28%, respectively) and 18:1n-9 and 22:6n-3 in phosphatidylcholine (from ~15 to 10% and 24 to 18%, respectively). This suggests that these fish were not utilizing energy reserves in previously described ways and appear to rely more on other lipid classes or body tissues for overwinter survival than those analyzed in this study.

## 1. Introduction

High levels of overwinter mortality among young-of-the-year (YOY) fish are a common phenomenon among temperate species. Mortality in this young age class has been noted in both wild and laboratory trials with white perch (*Morone americana*) [1], northern populations of Atlantic silversides (*Menidia menidia*) [2], and in age-0 rainbow trout (*Oncorhynchus mykiss*) in both laboratory and lake experiments [3]. Similarly, the abundance of age-1 striped bass (*Morone saxatilis*) in the Miramichi and Hudson rivers has been connected to the severity of the winter that they experience as YOY fish [4,5]. Overwinter mortality in YOY fish is attributed to a combination of stressors [6], including predation [7], thermal stress [8], the presence of parasites or pathogens [9], and starvation due to lack of prey, low interest in food consumption, or dependency on energy reserves [10,11]. Though single independent stresses are infrequently lethal, when a combination of stressors is present, YOY fish need to respond adequately in order to survive [12].

Feeding frequency decreases when water temperatures cool [11,13], and many fish species either reduce or cease feeding activity throughout winter [14]. During periods of starvation, fish must rely on their endogenous energy reserves, primarily lipids, but are also known to utilize protein and glycogen [14,15,16]. Energetic demands are principally filled by the catabolism of fatty acids (FA) in triacylglycerols (TAG), which are stored in tissues as energy reserves, including the liver and white muscle [15,17,18]. Young-of-the-year fish in temperate waters experience a conflict between two different survival challenges relating to the size of these energy reserves prior to entering winter [19]. Early in their first year, metabolized energy is typically directed to somatic growth to limit the risk of predation [19]. However, at some point, energy metabolism must shift to increase stored energy as lipids since larger endogenous reserves are necessary to survive periods of starvation [19]. This evolved response to competing threats is well documented in YOY striped bass from the Hudson River estuary (New York, NY, USA), as YOY fish do not begin storing lipids for energy purposes until early September and extending into November [20]. This limits the resources available and the scale of energy reserves for winter, which is linked to the size-dependent mortality pattern seen in this and other species [1,5,10,21].

In addition to TAG, phospholipids (PL) are vital during times of thermal acclimation experienced by YOY fish overwinter since they are key in maintaining cellular structure and are only metabolized during times of extreme and prolonged food scarcity [17,22]. Phospholipids are made up of a variety of different classes, some of the most abundant being phosphatidylethanolamine (PE) and phosphatidylcholine (PC). Specifically, in the muscle tissue, these PL classes have demonstrated particular benefits to fish during the winter with respect to ensuring the structural integrity and function of tissues during times of low environmental temperatures [23]. During the winter, PL assist in ensuring proper fluidity and structure of an organism’s cell membranes during periods of cold temperatures [23,24,25]. One way of maintaining this fluidity is by changing the FA composition of phospholipid classes, such as PE and PC, within cellular membranes as temperatures change [23,25,26]. For instance, in response to a decrease in temperature, several studies have found an increase in total PE, in addition to an alteration in FA, such as 18:1/22:6, in a variety of warm- and cold-adapted marine fish [23,24,27].

Striped bass are a temperate anadromous and euryhaline fish native to rivers and coastal waters adjacent to eastern North America that spawn in rivers as far south as northern Florida to rivers emptying into the southern Gulf of St. Lawrence in the north [28,29]. In Nova Scotia (NS, Canada), it is among the species of interest for commercial cultivation as they have a historical commercial presence and can be grown in freshwater (FW). Unfortunately, similar overwinter mortality described above can be seen in this captive setting. For example, YOY striped bass have commonly suffered up to 100% overwinter mortality when stocked experimentally in FW ponds during the fall of their first winter [30].

The present study set out to investigate the role of lipids stored in body tissues in the overwinter survival of YOY striped bass stocked in FW ponds. Specifically, the objective was to determine the extent to which YOY striped bass in this setting were exhausting their lipid reserves prior to the end of the winter season. Since fish were not feeding throughout the winter, we hypothesized that the lack of food intake would be mostly reflected in the liver lipids (primarily TAG). More specifically, we hypothesized that total lipid (TL) and TAG would decrease in the liver because the YOY fish were relying on it for energy. In addition, we anticipated that changes in tissue composition due to adaptation to winter water temperatures would occur in the white muscle lipids, specifically in the PL. PE, PC, and their FA compositions were quantified overwinter to explore the hypothesis that the concentration of PE and specific FA, including 18:1n-9, 22:6n-3 and other monoenic and polyenic lipid species, would increase overwinter in the muscle tissue as an adaptation to thermal change. Consequently, we evaluated the concentration of TAG, PC, and PE in muscle tissue and TAG in the liver of YOY striped bass, as well as the FA profiles of each lipid class in fish collected in late fall (November) and mid-winter (March).

## 2. Materials and Methods

### 2.1. Experimental Fish and Diet

The experimental fish were produced from eggs spawned from domestic broodstock (Shubenacadie River origin, NS, Canada) in June 2016 at the Aquaculture Centre at Dalhousie University, NS, Canada [31]. On 20 September 2016, as part of a larger experiment, 280 YOY striped bass (mean body wt. 21 g) were distributed at random (70 fish per tank) among four tanks within a 12-tank recirculation system (tank capacity: 150 L).

A diet was developed based on the proximate analysis of two wild juvenile striped bass (Shubenacadie River, NS, Canada), based on the premise that they were consuming an ideal preparatory overwinter diet (Table 1). Whole-body composition analysis was completed by the Department of Agriculture Laboratory Services (Bible Hill, NS, Canada). The resulting formulated experimental diet (Table 1) contained an 8% fat portion containing two sources of lipid: fish oil (7% inclusion) and poultry fat (1% inclusion; full list of ingredients in Table 1). Ingredients were sourced from Northeast Nutrition Inc. in Truro, NS, Canada, and the diet (pellet diameter: 2 mm) was produced in September 2016 at Dalhousie University’s feedmill (Agricultural Campus, Bible Hill, NS, Canada). Lipid and FA analysis of the experimental diet was conducted by the Marine Lipid Lab at Dalhousie University (Sexton Campus, Halifax, NS, Canada) in November of 2016 (results provided in Supplementary Table S1).

Feeding of the prepared diet began 20 September 2016, and each tank of fish was hand fed two to four times daily to apparent satiation. The rearing temperature was 18–20 °C from 20 September to 27 October, then decreased by 2 °C every two days between October 27 and 31, then by 1 °C per day, reaching 7 °C on 11 November. The appetite of the fish decreased during this thermal acclimation period, and fish stopped feeding on November 8 at approximately 9 °C. Throughout, oxygen saturation was >80%, salinity was 0.9–9.8 ppt (mean: 5.5 ppt), and photoperiod simulated natural daylength (latitude 45° N).

Fish were transported to the experimental field site, North River Fish Farm Ltd., Upper North River, NS, Canada (NRFF; 45°29′53″ N, 63°12′31″ W), ca. 20 k from the Aquaculture Centre, on 11 November 2016. During transportation in insulated tanks, stocking density was ca. 1 fish/L, and dissolved oxygen was 70–80%. Two ponds (mean depth: 4.3 m), referred to as the Middle (~8000 m^2^ surface area) and Lower (~6000 m^2^) ponds, were included in this specific study. There was water exchange from the Middle to the Lower pond, with a maximum flow rate of 120 L min^−1^ during the experiment. Each of the two ponds contained three 1 m^3^ cages stocked with 30 fish (mean body weight 46.4 g and fork length 14.3 cm) fed the experimental diet.

Water temperature and dissolved oxygen concentration in each pond was monitored every 30 min with a HOBO data logger (Onset U26-001, Hoskin Scientific, Burlington, ON, Canada) placed at the same depth (2 m) as the floor of the experimental cages. In addition, temperature was measured during sampling with a calibrated handheld meter (YSI Professional Plus, Hoskin Scientific, Burlington ON, Canada).

### 2.2. Sample Selection for Lipid Analysis

Initial pre-winter samples of fish (*n* = 5) were taken on November 15 from fish remaining at the lab in tanks with water temperatures of ca. 7 °C after other fish had been transferred to ponds. Late-winter samples were collected on 19 March 2017 from both ponds (*n* = 5 in each pond) by bringing cages partially to the surface, counting, and removing the surviving and dead fish. Survivors were euthanized (MS-222; 0.1–0.15 g L^−1^), and five fish per cage were selected at random for further lipid analysis. Fork length (cm) and body weight (g) were recorded; liver and white muscle tissue (approximately 1–2 cm wide ventral to the dorsal fins, above the lateral line) was dissected, wrapped in aluminum foil, and stored at −80 °C.

### 2.3. Lipid Analytical Methods

TL was determined for both liver and white muscle tissue (<1 g liver and 1.5 g muscle) following a modified Folch et al. [32] method, using a 2:1 CHCl_3_:MeOH extraction [33]. TL was determined gravimetrically on a wet-weight basis. Individual lipid classes of liver and muscle TL (*n* = 5 from each pond) were separated and recovered using thin layer chromatography (TLC) on glass plates (20 cm × 20 cm covered in silica gel coating of 250 µm). TL samples in 0.1 mL dichloromethane (15 mg 0.1 mL^−1^), as well as TAG, PE, and PC standards, were applied to the plate. A solvent system of CHCl_3_:MeOH:acetic acid:water (50:30:8:3) was first used to separate polar lipids, leaving TAG at the origin. The plate was then dried and developed in hexane:diethylether:acetic acid (70:30:2) to separate TAG from other neutral lipids [34]. Visualization of individual lipid classes was accomplished by spraying the developed plate with dichlorofluorescein in ethanol (0.2% in 96% ethanol) and placing it under a UV light. Bands containing individual lipid classes were scraped from the plate and then placed in appropriate solvents to solubilize the lipids (TAG: CHCl_3_; PE/PC: MeOH:CHCl_3_, 2:1). Solvent was evaporated under a nitrogen stream in a 25–30 °C water bath, and samples were then dissolved in dichloromethane and stored at −30 °C.

To determine the fatty-acid composition of liver and muscle TAG, as well as muscle PE and PC, fatty-acid methyl esters (FAME) were prepared by transmethylation of lipids using sulphuric acid as a catalyst with heating at 100 °C for an hour [33,35]. FAME were analyzed with gas chromatography (GC) using a Bruker 436 GC equipped with a DB-23 column (50%-cyanopropylmethylpolysiloxane; 30 m, 0.25 mm ID, 0.25 µm film thickness), using helium (flow rate 1 mL min^−1^) as carrier gas. Split injection (1/100) at 250 °C was used with flame ionization detection (280 °C with argon as make-up gas). The oven temperature was initially held at 150 °C for two minutes and then ramped at a rate of 5 °C min^−1^ until it reached 220 °C, at which point it was held for three minutes for a total runtime of 19 min. FA names are expressed in shorthand notation as A:Bn-C, where A represents the number of carbon atoms, B the number of double bonds, and C the position of the first double bond relative to the terminal methyl group.

High-performance liquid chromatography (HPLC) was utilized to quantify lipid classes (TAG, PE, and PC) and confirm results from the TLC procedure. A YMC-Pack PVA-SIL-NP column (polyvinyl alcohol phase, 150 mm × 4.6 mm ID, 5 µm particle size) with evaporative light-scattering detector (ELSD) was used. The detector was held at 42 °C with N_2_ at 3.5 barr. TAG and PL were quantified separately, following methods in Jones et al. (2012). The TAG method employed hexane:ethyl acetate (98.8:1.2) and isopropanol:MeOH:water (3:3:1) as mobile phases, while the PL method used ethyl acetate with 0.1 % acetic acid, in addition to isopropanol:MeOH:water (3:3:1; details in Supplementary Tables S2 and S3). Only TAG was determined in liver tissue; all three lipid classes were determined in the muscle tissue. All values were expressed on a wet-weight basis.

### 2.4. Data Display and Statistical Analysis

Concentrations of individual lipid classes are expressed as mg g^−1^ wet weight. Lipid class FA is expressed as the mass percent of total FA identified. Outliers were first identified and removed using Dixon’s Q test. TL (mg g^−1^) and lipid class data (mg g^−1^) were transformed by taking the natural logarithm. The Shapiro-Wilk test was then used to test for a normal distribution of residuals; the Brown-Forsythe test was used to check for equal variance. ANOVA was used to compare the mean lipid data of initial samples in November to those in late winter, with pairwise post hoc (Tukey) tests performed as appropriate. Permutational multivariate analysis of variance (PERMANOVA) was applied to determine similarity in FA profiles among samples. When appropriate, pairwise comparisons were made, generating pseudo-t-statistics with a *p*-value generated by Monte Carlo sampling (sample size was insufficient for permutational analysis). Multidimensional scaling (MDS) was then applied to the FA proportional data to visualize patterns in the data, and similarity percentages (SIMPER) were used to identify FA contributing >5% of the dissimilarity between groups of samples. FA contributing greater than 5% to dissimilarity were then evaluated as above to test for normal distribution of residuals and equal variance before comparing means with ANOVA, followed by the Tukey post hoc test as appropriate. Statistical analysis was done by pond and reported as such throughout the paper.

## 3. Results

### 3.1. Survival, Temperature, and Dissolved Oxygen

Survival rates were high, with 89 % (*n* = 80) and 99 % (*n* = 89) of the total 90 fish stocked in the cages at the beginning of winter alive in the Lower and Middle ponds when sampled in March. Daily mean temperatures of both Lower and Middle ponds were the same through November, increasing from 6 °C when fish were first placed in the ponds to 8.3 °C, then cooling to 3.1 °C by the end of the month. Ice also began to form at the end of November. Thereafter, for the duration of ice cover, the Lower pond was significantly colder than the Middle pond by between 0.3 and 0.8 °C (ANOVA, *F*(1,300) = 16.8, *p* < 0.001). Temperatures rose slightly in the beginning of December but remained below 4 °C. From mid-December to the end of February, both ponds cooled slowly by ca. 0.2 °C per week, reaching their coldest temperatures on 27 February 2017, 1.6 °C and 1.2 °C in the Middle and Lower pond, respectively (Supplementary Figure S1). During March, they warmed to 3.2 °C and 2.6 °C, respectively, by 22 March.

Dissolved oxygen in both ponds ranged between about 5 and 11 mg L^−1^ overwinter (Supplementary Figure S2). Dissolved oxygen in the Lower pond was significantly higher than in the Middle pond by at least 1 mg L^−1^ through most of winter (ANOVA, *F*(1,300) = 129.4, *p* < 0.001). Temporal changes in dissolved oxygen in both ponds were highly significant (ANOVA, *F*(5,300) = 106.4, *p* < 0.001), falling progressively through January to less than 6 mg L^−1^ around 26 February 2017 and then abruptly increasing by more than 3 mg L^−1^ over two days. Given the different temperature and dissolved oxygen levels in the two ponds, fish were separated into two groups by overwintering pond for statistical analysis.

### 3.2. Total Lipid and Lipid Classes in the Liver and White Muscle Tissues

Liver TAG concentrations (mg g^−1^) did not change overwinter, with means remaining between 182 and 202 mg g^−1^ (Table 2). Near the end of the winter (March sampling), TL was composed almost entirely of TAG (95 and 93 % in Lower and Middle pond, respectively). Mean liver TL concentration showed a lower trend in samples taken from fish in March in comparison to samples taken in November, but the difference was not statistically significant (Table 2). However, for both liver TL and liver TAG, a post hoc power analysis indicated that a sample size ~4× larger would have been necessary to detect a large effect (*D* = 0.8), with power of 80% and alpha of 5%. The same is true for our results for muscle PC, where same size is insufficient to support our null effect. In contrast to the liver data, mean muscle total lipid (TL; mg g^−1^ tissue) concentration of fish sampled in March was significantly higher (39 and 51 mg g^−1^) than in November (16.2 mg g^−1^) (ANOVA, *F*(2,12) = 42.9, *p* < 0.017).

In the muscle, mean TAG was higher in the winter (21 and 14 mg g^−1^ in Lower and Middle ponds, respectively) than in initial samples (4 mg g^−1^) (ANOVA, *F*(2,12) = 29.0, *p* < 0.006). The same was seen in white muscle PE masses, as overwinter samples were higher when compared to initial samples (ANOVA, *F*(2,12) = 21.6, *p* < 0.006). Concentrations were 1.5–2 times greater in the winter samples in both the Lower and Middle ponds (Table 2). Muscle PC masses were unchanged in the muscle after overwintering (Table 2).

### 3.3. Fatty-Acid Profiles for Liver and Muscle TAG

**Figure 1:**
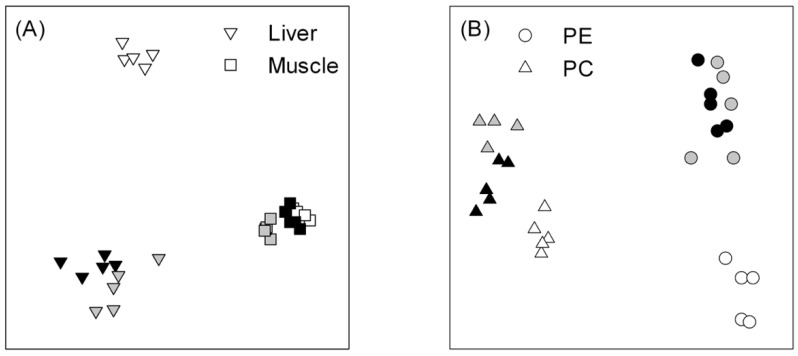
Multidimensional scaling plots of fatty acids (FA) in liver and white muscle tissue triacylglycerol (**A**), as well as in muscle phosphatidylethanolamine (PE) and phosphatidylcholine (PC) (**B**) of young-of-the-year striped bass sampled in November 2016 (white shapes) and March 2017 in Lower (black shapes) and Middle ponds (grey shapes).

**Figure 2:**
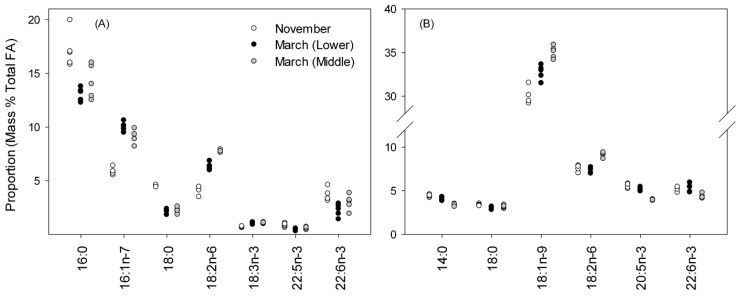
Proportions of fatty acids (FA) making greatest contributions to differences among samples in triacylglycerol of (**A**) liver (*n* = 5) and (**B**) muscle (*n* = 5). For the liver, within each FA, November samples are significantly different (Tukey’s test; *p* < 0.05) than all other samples for all FA except 22:6n-3, where the November samples are only different from the March (Lower pond) samples. In the muscle, FA proportions were different for all sampling times for 14:0, 18:1n-9, and 20:5n-3. For 18:0, November and March (Lower pond) were different; for 18:2n-6 and 22:6n-3, March (Middle pond) was different from both November and March (Lower pond).

The FA profile of the liver was dominated by 10 components (14:0, 16:0, 16:1n-7, 18:0, 18:1n-9, 18:1n-7, 18:2n-6, 20:1n-9, 20:5n-3, and 22:6n-3), with all present in proportions greater than 1 % and comprising > 90 % of the total profile (Supplementary Table S4). PERMANOVA indicated that FA profiles of liver TAG were different among the three samplings (Pseudo-*F*(2,12) = 16.6, *p* = 0.001); pairwise comparisons indicated that the November samples were distinct from the March samples (*p* = 0.001 for both), while samples from the Lower and Middle ponds in March were much more similar (*p* = 0.042). The MDS plot (Figure 1a) supported this interpretation, with March samples positioned close together and both well separated from the initial samples in November. SIMPER indicated that seven FA were responsible for ca. 65–80% of the dissimilarity between samples collected in November and March (Figure 2a), with all FA proportions, except 22:6n-3, significantly different in November and March samples (ANOVA with Tukey’s post hoc test with *p* < 0.02 for all).

The FA profile of the muscle was dominated by the same FA as the liver (Supplementary Table S5); however, overall, the TAG FA profiles in the muscle were much more similar to each other, relative to the liver, and positioned close together in the MDS plot (Figure 1a), although PERMANOVA still indicated that samples were different (Pseudo-*F*(2,12) = 24.5, *p* = 0.001), with pairwise comparisons suggesting that all were distinct (*p* = 0.001). The individual FA contributing to the dissimilarity varied between groups much more than they had in the liver; therefore, six FA contributing at least 5% to the dissimilarity in all comparisons were selected for display and analysis by ANOVA (Figure 2b). Agreeing with the MDS plot, the differences in proportions of individual FA between groups were much less in the muscle than in the liver (Figure 2). Pairwise comparisons after ANOVA did not show a consistent pattern in muscle FA; three of the FA (14:0, 18:1n-9, and 20:5n-3) had different proportions in all samples, and for two others (18:2n-6 and 22:6n-3), November and March (Middle pond) were the same, and both differed from March (Lower pond). For 18:0, November and March (Lower pond) samples were different.

### 3.4. Fatty-Acid Profiles in Phospholipids (PE and PC Classes) in Muscle

**Figure 3:**
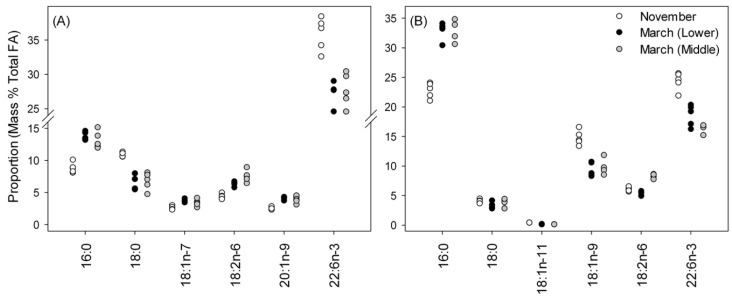
Proportions of fatty acids (FA) making greatest contributions to differences among samples in (**A**) phosphatidylethanolamine (PE; *n* = 5) and (**B**) phosphatidylcholine (PC; *n* = 5) in muscle. For all FA in PE, November samples are different (Tukey’s test; *p* < 0.05) from both March samples. For PC, November samples are different from both March samples for all FA, except 18:0, where samples were equivalent.

The same ten FA as identified in the liver also comprised > 90 % of all FA in PE (Supplementary Table S6). November samples plotted separately from March samples (Figure 1b) and PEMANOVA supported this difference (Pseudo-*F*(2,12) = 16.9; *p* = 0.009); however, the pairwise comparisons did not indicate a difference between PE FA profiles in the March samples. Six FA were responsible for 75 % of the dissimilarity between November and March samples (Figure 3a). All six FA showed the same pattern, with November samples significantly different from the March samples (ANOVA, followed by Tukey’s test, *p* < 0.001 for all).

For PC (Figure 3b), nine of the same ten FA were present in the largest proportions; in PC, 20:4n-6 was consistently present in higher proportions than 20:1n-9 (Supplementary Table S7). The MDS plot, again, shows clear separation of November and March samples with PERMANOVA, indicating that the difference was significant (Pseudo-*F*(2,12) = 14.4, *p* < 0.001); pairwise comparisons showed that FA profiles of March samples were significantly different from each other (*p* = 0.02) and the November samples (*p* = 0.001). Similar to PE, ANOVA indicated significant differences in all six FA identified by SIMPER as contributing to the dissimilarity among sample types. Tukey’s test showed that all November samples were different from both March samples (*p* < 0.02) for all six FA, except 18:0, where proportions were the same in all samples. The pattern for 18:2n-6 was unusual compared to the other FA in PC and PE in that the November sample had an intermediate proportion between the two March samples.

## 4. Discussion

This study consisted of a combination of two stressors, thermal acclimation and food scarcity, that a temperate YOY striped bass will encounter during their first winter. The fish included in this study were exposed to a natural winter environment and therefore experienced food scarcity and low water temperatures. It has been suggested that a combination of stressors, as opposed to a single one, is the cause of large mortality events in YOY temperate fish [6,36]. In the context of both of the stresses highlighted in this paper, lipids may play a role in survival; however, different classes of lipids fill specific adaptation niches, as discussed below.

### 4.1. Thermal Acclimation Overwinter

In the context of thermal acclimation, the typical role of PL is to maintain fluidity and structure of an organism’s cells. It is common to observe an increase in PE concentrations in tissues, such as the brain and liver of fish [23,24,37], as exhibited by the overwintering YOY striped bass. An increase in conic-shaped PE, in comparison to cylindrical-shaped PC, is thought to be one of the ways cellular membranes succeed in increasing fluidity during thermal changes or periods of cold temperatures [23,24,37,38]. Thus, these higher concentrations of PE likely point to a particular thermal acclimation mechanism utilized at the time of sampling by YOY striped bass in our trial.

The proportions of 18:0 and 22:6 in PE and 18:1 and 22:6 in PC were lower in March when compared to initial November samples. The general reaction of PL to a temperature reduction is to increase these FA, as seen in various warm- and cold-adapted fish species [23,24]. Reconstruction of the lipid structures in phospholipid bilayers occurs in colder temperatures as an adaptation to maintain cell fluidity [24,39]. In many FW and marine fish, the phospholipid classes, including PE and PC, contained higher proportions of specific monoenic and polyenic FA after adaptation to colder water temperatures ranging from ca. 5–10 °C [23]. This increase in monoenic/polyenic species (such as 18:1/22:6) seems to be a universal response of phospholipid remodelling of liver, brain, and gill samples of multiple cold-water- (5–10 °C) adapted FW and marine fish species when compared to warm-water- (20–27 °C) adapted species [23,24]. This phenomenon was explored by Buda et al. [38], as they observed an accumulation of monounsaturated/polyunsaturated FA in brain phospholipids of carp (*Cyprinus carpio*) when acclimatized to 5 °C water [38]. In that work [38], Buda et al. presented evidence that this observed change was due to the direct effect of temperature on the phospholipids since the fish had previously ceased feeding during the trial as temperatures decreased to below 10 °C. Although these studies suggest that an increase in monoenic/polyenic FA species occurs in colder temperatures, it is difficult to make comparisons with the present work since we only examined the effect of temperature on the FA profiles in the muscle.

Even so, the results of the muscle PL FA provided an interesting finding as the response of those lipids during cold-water adaptation was the opposite of that suggested by the literature. The lower proportion of both 22:6 and 18:0 in PE and 22:6 and 18:1 in PC overwinter does not seem like a coincidence. It appears those FA are being selectively mobilized or catabolized; however, the reason for this remains unclear. We also observed a significant increase in 16:0, a saturated FA, in both PL classes in the muscle tissue. Multiple components, including cholesterol, proteins, and the composition of the phospholipid class, can all impact the fluidity of a cellular membrane [40]; therefore, an increase in saturated FA in the muscle could indicate that another component or lipid class was assuming the role of regulating membrane fluidity.

It may be that the higher concentration in muscle TAG in March samples is a response to thermal stress and the decrease in temperature during the winter. A rise in TAG was observed in the muscle tissue of temperate eelpout (*Zosrces viviparous*) in relation to a decrease in PL and CHOL during a laboratory study comparing various changes in lipid concentrations in liver and muscle at different temperatures [41]. This accumulation of TAG was at its highest in *Z. viviparous* muscle at 4 °C compared to fish held at 6, 12, and 18 °C [41]. The increase was attributed to a shift to synthesis of storage lipids, or TAG, as water temperatures decrease [41]. However, in that study, fish were being fed for the duration of the four-month study, and it is therefore impossible to eliminate the possibility that the increase in TAG was facilitated by the consumption of dietary lipids. Observed higher concentrations of muscle TAG seems counterintuitive but may be filling some unknown role in adaptation to temperature.

### 4.2. Food Scarcity

Fish were not fed during the length of the overwinter trial, and at the time of sampling, fish had been without offered food for 131 days. From previous laboratory trials and observations, striped bass reared at the Dal-AC following the same procedures as this work show little interest in offered feed once temperatures are reduced to 5 °C (Duston et al., unpub. data). Nonetheless, there is evidence of Hudson River striped bass YOY cohorts feeding at a reduced rate throughout the months of five consecutive winters in temperatures ranging from 2 to 10.4 °C [42]. Striped bass from this population typically consume benthic invertebrates (predominantly *Gammarus* sp.) and small fish prey during the winter [42]. Therefore, while unlikely, we cannot eliminate the possibility of opportunistic food consumption throughout the winter.

During times of prolonged food deprivation or scarcity, energetic demands of an organism are satisfied by the oxidation of lipids, predominantly TAG [17]. Our findings, with liver TAG concentration remaining constant and higher TAG concentrations in the muscle of overwintering striped bass, are at odds with much prior research that indicates that various temperate species, including Atlantic silverside, rainbow trout, and white bass (*Morone chrysops*), experience a winter deficit or decrease in energy reserves throughout the winter [2,3,43]. Striped bass have also demonstrated this winter deficit. Hurst and Conover [20] report that Hudson River YOY striped bass experienced a loss of up to 21% of their total energy content in addition to 50% depletion of neutral lipid (storage lipids/TAG) over several winters (1993–1998) when winter-collected samples were compared to pre-winter samples. We were able to identify one study that supports an increase in total hepatic (liver) lipid in striped bass when acclimated to colder temperatures. Specifically, Stone and Sidell [44] determined that the TL in the liver was much higher in fingerling striped bass from the Hudson River population when acclimated to 5°C compared to 25 °C, with TL making up 40% and 25% of the liver dry weight, respectively. Stone and Sidell [44] also reported that carbohydrates comprised a much higher proportion of dry mass in the liver in warm-, rather than cold-, acclimated fingerlings (7 vs. 18%). This led to the conclusion that at colder temperatures, carbohydrates function as the primary source of energy in the liver, as opposed to lipids [44]. Therefore, the YOY striped bass in the present work may have been sparing TAG and catabolizing carbohydrates in their place for energy requirements during periods of cold temperatures.

The significantly higher concentrations of TL we found in the muscle in March could be the result of two possible changes in the tissue: (1) a proportionately greater reduction in another component in the muscle; or (2) a real increase in lipid. A slight increase in the proportion of muscle lipid relative to total tissue mass was observed in starved adult carp overwinter placed in monitoring net cages (Lake Kasumingaura, Japan) [45]. This was the result of a decrease in muscle protein when fish were stocked in lake water with winter (water) temperatures that ranged from 2.2 to 11.5 °C for a period of 128 days from November to March. Lipid concentrations in the visceral fat also decreased in these fish; therefore, the authors concluded that adult carp predominantly utilize muscle protein and visceral lipid as energy sources during winter starvation compared to muscle lipid [45]. However, a decrease in water content in the tissue could also have the same effect. We did not determine the proximate composition of either tissue, so are unable to confirm which component might have decreased to cause the increase in lipid. The possibility of the higher concentrations of TL after the winter in the YOY striped bass being due to a real increase in lipid deposition in the tissue seems unlikely. Fish that are able to feed overwinter are able to maintain their supply of lipid reserves and even show an increase in growth [46]. However, we assume fish were not feeding for the duration of our overwinter trial, and thus, there is no source of additional TAG and TL to deposit in muscle.

Although we did not analyze the visceral fat when sampling, we did observe the presence of visceral fat within the body cavity of the fish. It may be that YOY striped bass rely on the energy reserves present in this area of the body more so than that in the liver and muscle. In gizzard shad (*Dorosoma cepedianum*), liver is thought to be an “emergency reserve” that is only drawn upon during times of extreme need, while visceral fat serves as the main source of TAG supporting energetic needs [47,48]. Fetzer et al. [48] investigated the adaptations of gizzard shad during starvation and cold-temperature stress and concluded that fish were perhaps unable to access their energy reserves in the visceral tissue. In response to this, gizzard shad included in the Fetzer et al. [47] trial needed to turn to their emergency reserves in the liver, and as a result, were functioning as if they had exhausted their energy reserves [47]. Considering this, it may be that the situation in the ponds during the March sampling events was not dire enough for the YOY striped bass to require drawing on their lipid reserves found in the liver. Instead, up until the point of sampling, they may have been primarily utilizing reserves from their visceral fat to counteract the effects of not consuming food overwinter. Future work should also measure viscerosomatic indices, as well as viscera lipids, to evaluate this possibility.

Arctic charr (*Salvelinus alpinus*) and brook trout (*Salvelinus fontinalis*) were also placed in NRFF’s Lower and Middle pond from December 2016 to April 2017. Although these fish were left until the ice thawed in April, all fish (*n* = 15 per species in each pond) were alive at the time of sampling. Liver and muscle tissues were not analyzed for their TL or lipid class content; however, we observed an exhaustion of all visceral fat within the body cavity of the fish. This depletion of visceral fat reserves suggests that salmonids and striped bass adapt to overwinter stresses (food deprivation and cold-water thermal stress) in different ways, as visceral fat remained in the body cavity of YOY striped bass overwinter (when sampled in March). If liver and muscle tissues are the last resorts used by overwintering YOY striped bass to meet energetic demands, and given we only sampled survivors, it is possible that the TAG in these tissues had not yet started to be metabolized by fish sampled during our study. It would have been beneficial to sample fish as they were dying in order to determine if moribund fish were using alternative sources of reserves. This was not possible in the present study as fish could not be observed through the ice covering the ponds.

Furthermore, of the six FA that demonstrated significant changes in the liver tissue over the winter, the increases in proportions of 18:2n-6 and 18:3n-3 are among the most interesting. Several species of marine fish, including red and black sea bream (*Pagrus major* and *Spondyliosoma cantharus*), opaleye (*Girella nigricans*), and striped mullet (*Mugil cephalus*) [49], have only a limited ability to convert 18:3n-3 into 22:6n-3 during starvation. Hixon et al. [50] also discussed the very limited ability of the marine Atlantic cod (*Gadus morhua*) to desaturate and elongate 18:3n-3 when fed experimental diets containing high proportions of n-3 FA. In general, marine fish have very low ability to convert 18:3 to long-chain polyunsaturated FA (LC PUFA) due to potential low activity or deficiencies of 5-desaturase and therefore require the inclusion of FA, such as eicosapentaenoic (EPA) and docosahexaenoic (DHA) acids, in their diets [51,52,53]. Indeed, Webster and Lovell [54] reported that striped bass larvae seemed incapable of elongation and desaturation of FA chains into long-chain essential FA when fed a diet of brine shrimp containing high levels of linolenic acid (18:2n-6). In contrast, FW species are able to meet their LC PUFA requirements by elongation and desaturation of dietary C18 FA, specifically 18:3n-3 and 18:2n-6, to form 20:5n-3, 22:6n-3, and 20:4n-6 [52,53,55]. It may be that although typically classified as FW fish, YOY striped bass have lipid metabolism more similar to marine fish. This slight to non-existent ability to transform 18 C FA into essential FA (20:5n-3 and 22:6n-3) amongst marine species could support the observed high level of 18 C FA in the liver of YOY striped bass, explained by the retention or accumulation of essential 18 C FA in the face of starvation. Dietary requirements for striped bass in culture are not well known; however, studies investigating hybrid striped bass (*Morone chrysops* × *Morone saxitilis*) have indicated that their nutritional needs reflect those of marine fish [54,56,57,58,59]. Thus, they should have little capacity to use those precursors to form long-chain essential FA, leading to an accumulation of 18:2n-6 and 18:3n-3, as seen in March samples. Further, Barry and Trushenski [60] have recently reported that hybrid striped bass diets should include n-3 and n-6 LC PUFA in order to make sure essential fatty-acid requirements are met since they are unable to do so with only C18 PUFA. This finding is important to consider when formulating diets for striped bass to ensure they are provided with the appropriate essential FA. Future research is needed to know the full extent of dietary FA required by striped bass, and trials monitoring their ability to convert C18 into the necessary LC PUFA would be a step towards accomplishing this.

In summary, YOY striped bass were unique in their utilization of lipid reserves in the muscle and liver when held overwinter in FW ponds. The observed higher concentration overwinter in muscle TAG is a possible adaptation process by which YOY striped bass combat winter temperatures, although it is unclear how this adaptation might be of benefit. PE and PC FA did not respond as expected to thermal adaptation. Perhaps a way forward is to determine the tissues that striped bass YOY rely on for energy sources during the winter, as our results are inconclusive. Further investigation into the overwinter adaptations of Shubenacadie River population-derived YOY striped bass is necessary. If lipids were not being used as the primary source of energy, then another constituent of the liver and muscle must have been; however, we were unable to identify the constituent.

## Figures and Tables

**Table 1:** Complete ingredient list and inclusion by mass for experimental diets fed to young-of-the year striped bass (*Morone saxatilis*) during the laboratory feeding period (20 September–8 November 2016) prior to transfer to freshwater ponds.

Ingredient Name	g Per kg
Fish Meal	500
Empyreal	125
Blood Meal	111.5
Ground Wheat	100
Fat	80
Wheat Gluten	50
Dicalcium Phosphate	20.5
Lysine HCI	5
Choline Chloride	3
Vitamin/Mineral Premix ^1^	2.5
Special Premix ^2^	2.5
Total	1000

^1^ Special premix contained: vitamin E 250 mg, vitamin C 200 mg, astaxanthin 60 mg, selenium 0.22 mg, and wheat shorts 1988 mg (per kg). ^2^ Vitamin/mineral premix contained: manganese 125 mg, iron 84 mg, zinc 77.5 mg, copper 2.5 mg, iodine 7.5 mg, vitamin A 5000 IU, vitamin D 4000 IU, vitamin K 2 mg, vitamin B12 4 μg, thiamin 8 mg, riboflavin 18 mg, pantothenic acid 40 mg, niacin 100 mg, folic acid 4 mg, biotin 0.6 mg, pyridoxine 15 mg, inositol 100 mg, ethoxyquin 42 mg, and wheat shorts 1372 mg (per kg).

**Table 2:** Mean (±SD) total lipid and liver triacylglycerols (TAG), white muscle TAG, phosphatidylethanolamine (PE), and phosphatidylcholine (PC) concentrations (mg g^−1^ determined on a wet-weight basis) of young-of-the-year striped bass (*Morone saxatilis*) held in two freshwater ponds in November 2016 and March 2017.

Sample	November 2016	March 2017 (Lower Pond)	March 2017 (Middle Pond)
Total Lipid-Liver	236 ± 9 ^a^	201 ± 33 ^a^	213 ± 24 ^a^
Total Lipid-Muscle	16 ± 2 ^a^	51 ± 9 ^b^	39 ± 9 ^b^
Liver TAG	182 ± 9 ^a^	191 ± 35 ^a^	202 ± 16 ^a^
Muscle TAG	4 ± 1 ^a^	21 ± 6 ^b^	14 ± 5 ^b^
Muscle PE	2 ± 0.4 ^a^	4 ± 0.3 ^b^	3 ± 0.7 ^b^
Muscle PC	1 ± 1 ^a^	2 ± 0.2 ^a^	1 ± 0.2 ^a^

Within a row, values with different superscripts are significantly different (TL and TAG: *p* < 0.017; PE and PC: *p* < 0.006). *N* = 5 for all samples except for November TAG liver, where *n* = 4.

## Data Availability

All data are available from the authors upon request.

## Supplementary Materials

The following are available online at https://www.mdpi.com/article/10.3390/biom11111678/s1, Figure S1: Daily mean temperatures (°C) over the course of the experiment in the Middle and Lower ponds. Figure S2: Daily mean dissolved oxygen concentrations (mg L^−1^) over the course of the experiment in both the Middle and Lower ponds, Table S1: Proportions of fatty acids (FA; mass % of total FA identified) in the experimental diet used during the pre-winter feeding period from September 20 to the end of November 8 prior to transferring young-of-the-year striped bass (*Morone saxatilis*) to freshwater ponds at North River Fish Farms Ltd. for the duration of the winter, Table S2: High-performance liquid chromatography solvents and gradient program for triacylglycerols profile separation, Table S3: High-performance liquid chromatography solvents and gradient program for full phospholipid profile separation, Table S4: Proportions of individual fatty acids (FA; mass % of total FA identified) from liver triacylglycerols (TAG) included in statistical analysis. Samples were collected from young-of-the-year striped bass (*Morone saxatilis*) in November 2016 (initial samples) and March 2017 (overwinter samples). Table S5: Proportions of individual fatty acids (FA; mass % of total FA identified) from muscle triacylglycerols (TAG) included in statistical analysis. Samples were collected from young-of-the-year striped bass (*Morone saxatilis*) in November 2016 (initial samples) and March 2017 (overwinter samples). Table S6: Proportions of individual fatty acids (FA; mass % of total FA identified) from muscle phosphatidylethanolamine (PE) included in statistical analysis. Samples were collected from young-of-the-year striped bass (*Morone saxatilis*) in November 2016 (initial samples) and March 2017 (overwinter samples). Table S7: Proportions of individual fatty acids (FA; mass % of total FA identified) from muscle phosphatidylcholine (PC) included in statistical analysis. Samples were collected from young-of-the-year striped bass (*Morone saxatilis*) in November 2016 (initial samples) and March 2017 (overwinter samples.
