# Supplementary material for: Overwinter Changes in the Lipid Profile of Young-of-the-Year Striped Bass (Morone saxatilis) in Freshwater Ponds
Source: Biomolecules. 2021 Nov 11;11(11):1678. doi: 10.3390/biom11111678 (PMC8615886; doi:10.3390/biom11111678)
Supplement: Supplementary file 1 [file biomolecules-11-01678-s001.zip › biomolecules-1411674-supplementary.pdf]

Supplementary Material

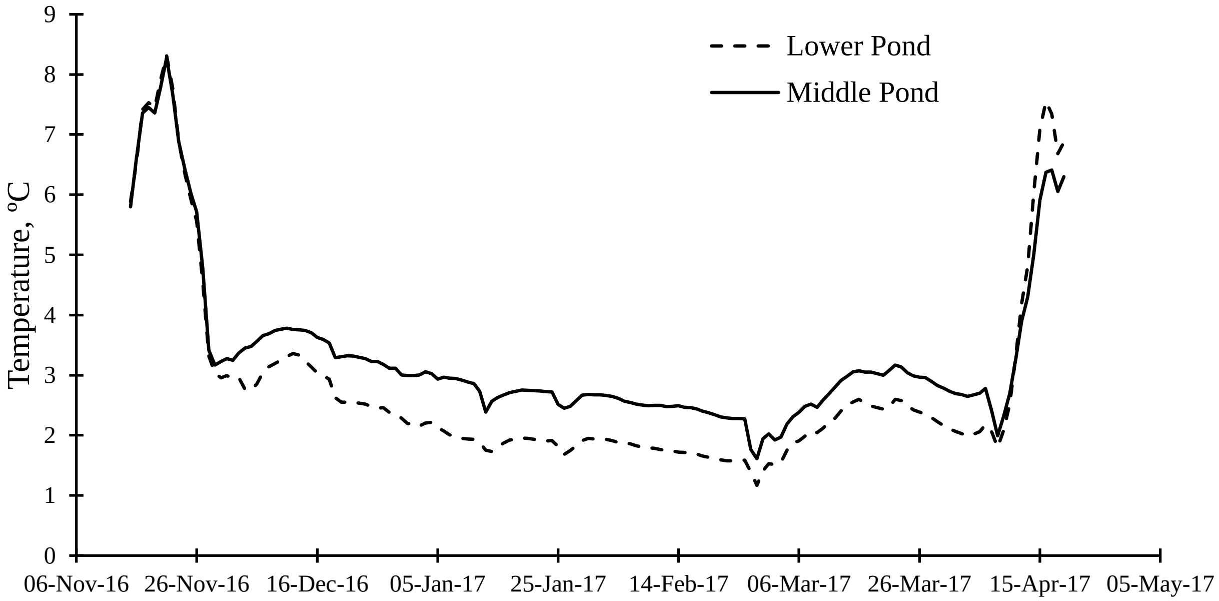

Figure S1. Daily mean temperatures (°C) over the course of the experiment in the Middle and Lower ponds.

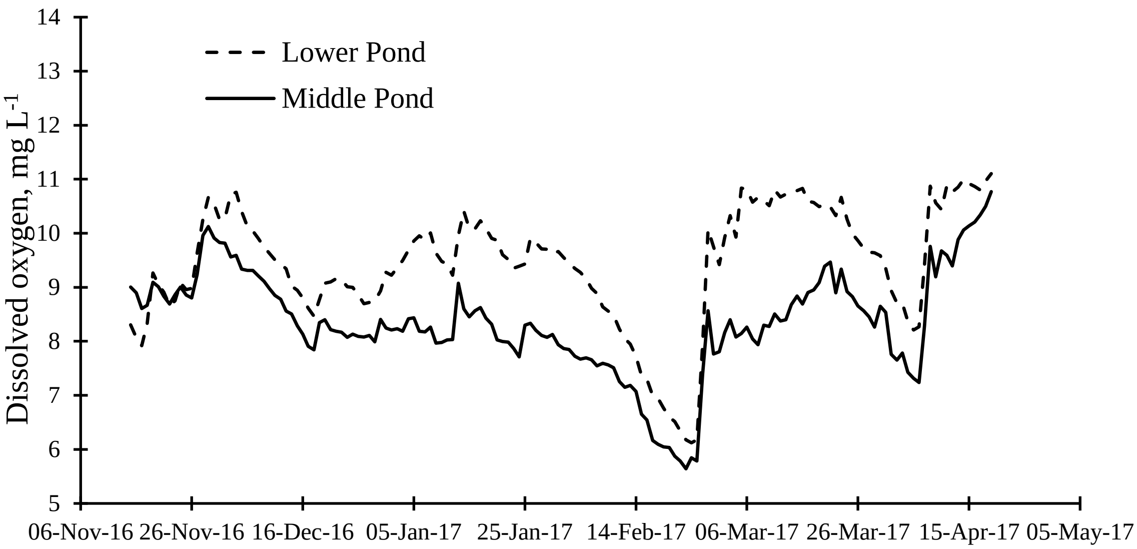

Figure S2. Daily mean dissolved oxygen concentrations (mg L^{-1}) over the course of the experiment in both the Middle and Lower ponds.

Table S1. Proportions of fatty acids (FA; mass % of total FA identified) in the experimental diet used during the pre-winter feeding period from September 20 to the end of November 8 prior to transferring young-of-the-year striped bass (*Morone saxatilis*) to freshwater ponds at North River Fish Farms Ltd. for the duration of the winter.

FA	%	FA	%	FA	%
**12:0**	0.1	**16:3n-4**	0.97	**20:1n-7**	0.16
**13:0**	0.03	**17:01**	0.18	**20:2n-9**	0
**i-14:0**	0.03	**16:4n-3**	0.06	**20:2n-6**	0.17
**14:0**	6.3	**16:4n-1**	0.77	**20:3n-6**	0.19
**14:1n-9**	0.09	**18:0**	3.39	**20:4n-6**	0.89
**14:1n-7**	0.02	**18:1n-13**	0.03	**20:3n-3**	0.06
**14:1n-5**	0.06	**18:1n-11**	0.06	**20:4n-3**	0.57
**i-15:0**	0.14	**18:1n-9**	17.34	**20:5n-3**	9.61
**ai-15:0**	0.04	**18:1n-7**	2.58	**22:0**	0.19
**15:0**	0.4	**18:1n-5**	0.08	**22:1n-11**	0.57
**i16:0**	0.06	**18:2n-7**	0.07	**22:1n-9**	0.17
**16:0**	18.15	**18:2n-6**	10.5	**22:1n-7**	0.06
**16:1n-11**	0.28	**18:2n-4**	0.23	**22:2n-6**	0.01
**16:1n-9**	0.21	**18:3n-6**	0.18	**21:5n-3**	0.4
**16:1n-7**	7.25	**18:3n-4**	0.26	**23:0**	0.05
**16:1n-5**	0.15	**18:3n-3**	1.95	**22:4n-6**	0.15
**17:1(a)**	0.03	**18:3n-1**	0.1	**22:5n-6**	0.28
**i-17:0**	0.13	**18:4n-3**	1.14	**22:4n-3**	0.05
**16:2n-6**	0.14	**18:4n-1**	0.18	**22:5n-3**	1.56
**ai-17:0**	0.11	**20:0**	0.27	**24:0**	0.16
**17:1(b)**	0.14	**20:1n-11**	0.11	**22:6n-3**	7.67
**16:2n-4**	0.87	**20:1n-9**	0.94	**24:1n-9**	0.34
**15:0**	0.36				

Table S2. High performance liquid chromatography solvents and gradient program for triacylglycerols profile separation.

Time (min)	Solvent A (%)	Solvent B (%)	Flow rate (mL/min)
1.5	100	0.0	1.5
2	60	40	1.5
6	25	75	1.5
7	100	0.0	1.5
15	100	0.0	1.5

Solvent A: hexane: ethyl acetate (98.8:1.2)

Solvent B: isopropanol: methanol: water (3:3:1) with 0.1 % acetic acid and *0.005% triethylamine.

Table S3. High performance liquid chromatography solvents and gradient program for full phospholipid profile separation.

Time (min)	Solvent A (%)	Solvent B (%)	Solvent C (%)	Flow rate (mL/min)
8	0.0	100	0.0	1.5
9	0.0	50	50	1.5
15	0.0	15	85	1.5
20	0.0	0.0	100	1.3
29	0.0	100	0.0	1.0
31	0.0	100	0.0	1.0

Solvent A: hexane: ethyl acetate (98.8:1.2),

Solvent B: isopropanol: methanol: water (3:3:1) with 0.1 % acetic acid and *0.005% triethylamine.

Solvent C: ethyl acetate with 0.1% acetic acid.

Table S4. Proportions of individual fatty acids (FA; mass % of total FA identified) from liver triacylglycerols (TAG) included in statistical analysis. Samples were collected from young-of-the-year striped bass (*Morone saxatilis*) in November 2016 (initial samples) and March 2017 (overwinter samples).

Sample ID #	Fatty acid proportions (%)												
	14:0	16:0	16:1n-7	18:0	18:1n-9	18:1n-7	18:2n-6	18:3n-3	20:1n-9	20:2n-6	20:5n-3	22:5n-3	22:6n-3
6 – Low. L	1.99	13.07	10.38	1.79	39.44	4.02	5.9	0.96	7.99	1.17	2.04	0.55	2.81
7 – Low. L	1.99	12.01	9.93	1.79	41.97	4.10	6.24	0.96	8.19	1.17	1.78	0.35	1.90
8 – Low. L	2.29	13.17	9.38	2.04	38.61	4.00	6.55	1.09	6.77	1.02	2.23	0.47	2.27
9 – Low. L	1.89	12.65	9.57	2.27	39.11	4.28	5.70	0.93	7.17	1.03	2.04	0.48	2.53
10 – Low. L	2.02	12.00	9.09	2.16	40.44	4.36	6.00	0.88	8.76	1.17	1.44	0.30	1.36
16 – Mid. L	1.92	14.75	8.36	2.03	39.45	3.84	7.28	0.93	6.27	1.06	1.34	0.40	1.82
17 – Mid. L	1.83	13.50	8.56	2.21	39.95	3.69	7.55	1.08	6.44	1.24	1.86	0.55	3.00
18 – Mid. L	1.75	11.95	9.43	1.77	37.96	3.78	7.56	1.09	7.44	1.44	1.78	0.67	3.68
19 – Mid. L	1.63	12.31	8.90	2.12	41.56	3.84	7.28	1.00	5.81	0.98	1.59	0.54	3.07
20 – Mid. L	1.81	12.66	8.54	2.09	39.90	3.775	7.955	1.12	6.975	1.44	1.65	0.42	1.995
1 – Initial L	1.87	19.62	5.67	4.46	41.41	2.97	4.32	0.62	7.02	0.89	1.55	0.64	3.13
2 – Initial L	2.08	15.49	5.41	4.43	39.83	3.48	4.05	0.71	8.01	0.98	2.46	1.06	4.52
3 – Initial L	2.16	16.62	6.29	4.48	40.71	3.24	3.44	0.60	9.42	0.89	1.86	0.75	3.10
4 – Initial L	2.24	16.70	5.57	4.53	40.19	3.55	4.04	0.68	8.56	1.05	2.05	0.84	3.28
5 – Initial L	2.27	15.67	5.76	4.31	39.35	3.47	4.33	0.74	8.62	1.14	2.41	0.95	3.73

*Low. L: Lower pond liver TAG samples; Mid. L: Middle pond liver TAG samples; Initial L: initial sampling event liver TAG samples.

Table S5. Proportions of individual fatty acids (FA; mass % of total FA identified) from muscle triacylglycerols (TAG) included in statistical analysis. Samples were collected from young-of-the-year striped bass (*Morone saxatilis*) in November 2016 (initial samples) and March 2017 (overwinter samples).

Sample ID #	Fatty acid proportions (%)												
	14:0	16:0	16:1n-7	18:0	18:1n-9	18:1n-7	18:2n-6	18:3n-3	20:1n-9	20:2n-6	20:5n-3	22:5n-3	22:6n-3
1 – Low. M	4.08	18.20	7.11	2.99	30.94	2.51	7.18	1.18	3.33	0.49	4.83	1.20	4.61
2 – Low. M	3.89	18.54	7.06	3.09	32.69	2.45	6.78	1.09	3.60	0.46	4.80	1.17	4.74
3 – Low. M	4.06	18.15	6.82	2.89	32.18	2.52	7.25	1.18	3.25	0.44	5.03	1.33	5.31
4 – Low. M	4.17	18.30	7.16	2.73	30.59	2.62	7.49	1.20	3.37	0.51	5.29	1.31	5.28
5 – Low. M	3.73	18.21	6.92	2.86	32.06	2.56	6.89	1.07	3.69	0.50	5.01	1.34	5.78
11 – Mid. M	3.23	18.03	6.31	2.72	31.86	2.32	8.55	1.17	3.11	0.52	3.71	0.94	3.82
12 – Mid. M	3.18	17.91	6.55	2.96	33.94	2.40	8.78	1.18	3.44	0.58	3.78	0.97	4.19
13 – Mid. M	3.23	17.73	6.32	2.98	31.99	2.36	8.68	1.18	3.60	0.59	3.70	0.96	4.12
14 – Mid. M	3.05	18.15	6.34	3.24	34.36	2.35	8.31	1.13	3.03	0.48	3.70	1.09	4.58
15 – Mid. M	2.92	16.94	6.10	2.96	32.28	2.41	8.63	1.16	3.27	0.58	3.61	0.99	3.89
1 – Initial M	4.47	19.46	6.95	3.22	28.47	2.65	7.70	1.35	3.17	0.54	5.67	1.29	5.30
2 – Initial M	4.11	19.84	6.89	3.45	30.83	2.56	6.89	1.21	3.47	0.51	5.12	1.17	4.69
3 – Initial M	4.26	19.30	6.94	3.29	29.46	2.60	7.31	1.23	3.84	0.48	5.26	1.19	5.05
4 – Initial M	4.47	19.46	6.95	3.22	28.47	2.65	7.70	1.35	3.17	0.54	5.67	1.29	5.30
5 – Initial M	4.34	19.16	6.98	3.17	28.76	2.63	7.60	1.33	3.46	0.56	5.59	1.29	5.32

*Low. M: Lower pond muscle TAG samples; Mid. M: Middle pond muscle TAG samples; Initial M TAG: initial sampling event muscle TAG samples.

Table S6. Proportions of individual fatty acids (FA; mass % of total FA identified) from muscle phosphatidylethanolamine (PE) included in statistical analysis. Samples were collected from young-of-the-year striped bass (*Morone saxatilis)* in November 2016 (initial samples) and March 2017 (overwinter samples).

Sample ID #	Fatty acid proportions (%)														
	14:0	16:0	16:1n-7	18:0	18:1n-9	18:1n-7	18:2n-6	18:3n-3	20:1n-9	20:2n-6	20:4n-6	20:5n-3	22:5n-6	22:5n-3	22:6n-3
1 – Low. M	0.76	12.92	1.88	6.93	11.08	3.39	5.67	0.62	4.00	1.01	2.90	12.53	0.79	3.06	27.11
2 – Low. M	0.98	14.07	2.25	5.35	12.78	3.58	6.13	0.67	4.17	0.97	2.35	12.56	0.69	2.77	23.70
3 – Low. M	0.60	14.03	1.52	5.36	10.86	3.99	6.59	0.65	3.65	1.07	2.39	11.91	0.87	3.15	28.42
4 – Low. M	0.77	13.17	1.82	5.48	11.13	3.65	6.28	0.65	4.09	1.05	2.46	13.11	0.82	2.83	26.98
5 – Low. M	0.63	13.10	1.75	7.84	11.03	3.65	6.42	0.56	4.14	1.12	2.67	11.07	0.83	3.15	27.28
11 – Mid. M	0.39	11.52	1.38	4.50	9.57	3.40	7.17	0.62	4.02	1.13	2.74	13.90	0.73	2.68	28.12
12 – Mid. M	0.71	14.89	1.71	7.96	11.35	2.64	6.34	0.58	3.07	0.85	3.15	12.92	0.72	2.63	26.02
13 – Mid. M	0.57	11.43	1.65	6.71	10.73	3.21	7.32	0.65	4.32	1.19	2.79	11.98	0.82	2.73	26.08
14 – Mid. M	0.53	13.37	1.70	6.00	12.17	4.01	8.64	0.70	3.82	1.20	2.61	11.65	0.72	2.88	23.72
15 – Mid. M	0.34	12.07	1.00	7.44	8.71	3.07	6.83	0.54	3.57	1.15	3.02	12.98	0.78	2.80	29.22
1 – Initial M	0.46	9.43	1.58	9.88	11.63	2.82	4.65	0.58	2.24	0.69	2.20	10.37	0.73	2.52	30.41
2 – Initial M	0.37	8.27	1.40	10.02	10.31	2.31	4.19	0.62	2.58	0.71	2.40	11.32	0.81	2.96	32.11
3 – Initial M	0.31	7.57	1.30	9.94	9.61	2.55	3.99	0.54	2.66	0.71	2.36	10.90	0.81	2.87	34.35
4 – Initial M	0.33	8.28	1.17	10.56	8.65	2.24	4.07	0.52	2.16	0.70	2.23	10.33	0.85	3.04	34.72
5 – Initial M	0.31	7.77	1.12	10.45	8.80	2.19	3.73	0.53	2.35	0.72	2.24	10.70	0.83	2.86	36.06

*Low. M: Lower pond muscle PE samples; Mid. M: Middle pond muscle PE samples; Initial PE: initial sampling event muscle PE samples.

Table S7. Proportions of individual fatty acids (FA; mass % of total FA identified) from muscle phosphatidylcholine (PC) included in statistical analysis. Samples were collected from young-of-the-year striped bass (*Morone saxatilis*) in November 2016 (initial samples) and March 2017 (overwinter samples.

**Sample ID #**	**Fatty acid proportions (%)**													
	**14:0**	**16:0**	**16:1n-7**	**18:0**	**18:1n-9**	**18:1n-7**	**18:2n-6**	**18:3n-3**	**20:1n-9**	**20:4n-6**	**20:5n-3**	**22:5n-6**	**22:5n-3**	**22:6n-3**
1 – Low. M	1.24	32.55	2.46	3.32	10.37	1.06	5.64	0.57	1.18	1.98	15.98	0.58	2.31	15.92
2 – Low. M	1.19	29.81	2.31	4.07	10.48	1.04	5.35	0.65	1.13	2.01	16.81	0.63	2.65	16.77
3 – Low. M	0.83	33.14	1.64	2.77	8.64	0.84	4.87	0.52	0.72	1.98	17.31	0.69	2.64	19.61
4 – Low. M	1.01	33.01	1.86	2.97	8.17	0.89	5.43	0.61	0.94	1.74	15.84	0.74	2.62	20.03
5 – Low. M	0.87	33.62	1.67	3.36	8.39	0.94	4.98	0.44	0.95	1.95	16.73	0.69	2.60	18.96
11 – Mid. M	1.09	34.51	2.46	2.27	9.04	0.83	8.44	0.73	0.85	1.59	14.83	0.60	2.54	15.62
12 – Mid. M	1.12	30.08	2.55	4.16	11.65	1.14	8.08	0.68	1.34	1.94	13.41	0.58	2.18	16.53
13 – Mid. M	1.10	34.31	2.42	2.78	9.61	0.83	8.51	0.68	1.12	1.73	14.78	0.57	2.34	15.01
14 – Mid. M	0.97	31.50	2.00	3.79	9.18	0.89	7.68	0.66	0.77	2.44	16.74	0.61	2.61	16.33
15 – Mid. M	0.95	33.27	1.95	4.36	8.36	0.84	8.40	0.65	0.83	2.20	14.35	0.61	2.35	16.58
1 – Initial M	0.80	22.78	2.51	4.42	16.34	1.25	6.14	0.66	0.93	1.95	13.40	0.71	1.85	21.57
2 – Initial M	0.60	20.78	2.32	4.09	15.10	1.20	5.88	0.62	0.99	2.22	14.79	0.80	2.02	24.33
3 – Initial M	0.69	21.61	2.29	3.76	14.18	1.17	5.58	0.65	0.91	2.22	14.43	0.76	2.04	25.28
4 – Initial M	0.82	23.56	2.37	3.98	13.87	1.33	6.41	0.74	1.01	1.86	12.45	0.75	2.09	23.56
5 – Initial M	0.72	23.45	2.14	3.61	13.17	1.16	5.73	0.67	0.91	2.07	14.15	0.74	1.98	25.03

*Low. M: Lower pond muscle PC samples; Mid. M: Middle pond muscle PC samples; Initial: initial sampling event muscle PC samples.
